# Use of cardiovascular magnetic resonance in the evaluation of a giant right atrial appendage aneurysm: a case report and review of the literature

**DOI:** 10.1186/s13104-017-3046-2

**Published:** 2017-12-04

**Authors:** Lojan Sivakumaran, Karl Sayegh, Emile Mehanna, Frank W. Sanchez, Jonathan Fields, Ricardo Cury

**Affiliations:** 1 Université de Montréal, 2900 Boulevard Edouard-Montpetit, Montreal, QC H3T 1J4 Canada; 2 McGill University Health Centre, 1650 Cedar Ave, Montreal, QC H3G 1A4 Canada; 3 Centre de recherche du Centre hospitalier de l’Université de Montréal, 900 St Denis, Montreal, QC H2X 0A9 Canada; 4 Miami Cardiac and Vascular Institute Baptist Hospital of Miami, 8900 N Kendall Drive, Miami, FL 33176 USA

**Keywords:** Right atrial appendage aneurysm, Cardiovascular magnetic resonance (CMR), Thrombus, Congenital heart anomaly

## Abstract

**Background:**

Right atrial appendage aneurysms are rare entities that may have significant clinical consequences. When co-existing with atrial fibrillation, patients are at risk of developing pulmonary or paradoxical systemic emboli.

**Case presentation:**

An elderly patient presented to medical attention with symptoms of acute diverticulitis. On abdominal computed tomography, a massively enlarged right atrial appendage aneurysm was discovered incidentally. The aneurysm caused marked compression of the right ventricle and contained an area of hypoenhancement concerning for an intraluminal thrombus. Gadolinium-enhanced cardiovascular magnetic resonance was performed and first-pass perfusion images demonstrated that the area of hypoenhancement was in fact poorly mixing blood. The patient was therefore managed medically.

**Conclusion:**

Right atrial appendage aneurysms are infrequently encountered cardiac abnormalities. In the literature, surgery has been offered to patients who are young, symptomatic, or have evidence of thrombotic disease, although whether this practice pattern is associated with superior clinical outcomes is unclear. In the present case, gadolinium-enhanced cardiovascular magnetic resonance imaging was used to exclude the presence of intraluminal thrombus in an elderly patient, which helped orient the patient’s treating team towards medical—rather than surgical—therapy.

**Electronic supplementary material:**

The online version of this article (10.1186/s13104-017-3046-2) contains supplementary material, which is available to authorized users.

## Background

Right atrial appendage (RAA) aneurysms are abnormal dilatations of the RAA that are associated with dysplasia and fibrosis of the atrial tissue [1, 2]. They are rarely reported in the literature and are less frequently encountered compared to other cardiac aneurysms [3]. A literature review published in 2014 found that the highest prevalence of RAA aneurysms was in the third decade of life, with cases noted from the perinatal period to 72 years of age [4]. Since this review was published, however, there have been several additional pediatric cases reported [5–9].

RAA aneurysms are typically asymptomatic and are often discovered incidentally. The most common clinical findings in patients with symptomatic RAA aneurysms are persistent atrial tachyarrhythmia [5, 10–14] and dyspnea [6, 15]. Less frequently encountered findings are outlined in Table 1. While RAA aneurysms have been found in patients with atrial septal defects [7, 10, 12, 13, 16], ventricular septal defects, spontaneous pneumothoraces [12], left atrial appendage aneurysms, and tricuspid/mitral regurgitation [11], there is currently no clear etiological relationship between RAA aneurysms and these pathologies.

**Table 1:** Clinical findings in patients with right atrial appendage aneurysms

Symptoms	Signs
Palpitations [5, 10, 14] Dyspnea [6, 15] Chest discomfort [8] Right heart failure [18]	Persistent atrial tachyarrhythmia [5, 10–14] Intermittent atrial tachyarrhythmia [8] Cyanosis [19]^a^ Clubbing [19]^a^ Mitral/tricuspid regurgitation [19]^a^

^a^Patient also had a left atrial appendage aneurysm

As RAA aneurysms are often diagnosed in the context of atrial fibrillation, the development of intraluminal thrombosis and subsequent pulmonary or paradoxical systemic embolism is of concern [17, 18]. Thrombus formation is as an indication in the literature for surgical resection of RAA aneurysms [17, 18]. However, the differentiation of an intraluminal thrombus from flow stasis is not always straightforward, especially when using echocardiography and contrast-enhanced computed tomography (CT) alone. We therefore present the use of cardiovascular magnetic resonance (CMR) to characterize a massive intraluminal hypodensity/filling defect in a patient with a RAA aneurysm found on CT.

## Case presentation

An elderly patient between 50–70 years of age (details obscured to protect patient privacy) presented to medical attention with increasing left lower quadrant pain and nausea over 3 days. Relevant past medical history was significant for atrial fibrillation, diabetes mellitus, and hypertension. Family history, medications, and allergies were non-contributory.

**Fig. 1:**
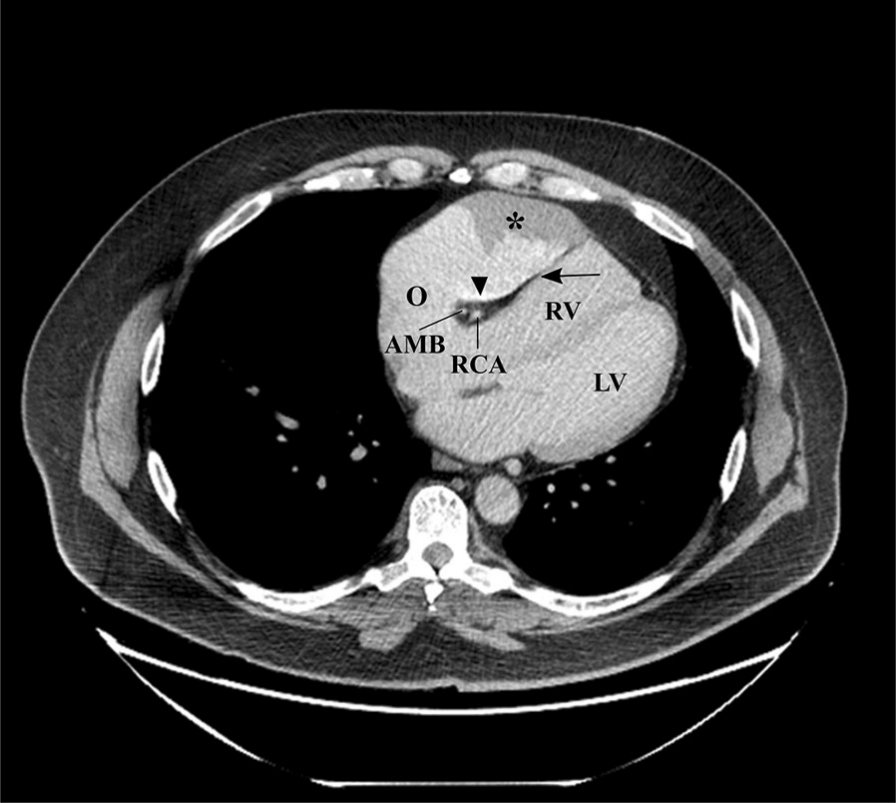
Computed tomography image showing the right atrial appendage aneurysm and an intraluminal filling defect. Axial computed tomography image (2.5 mm slice thickness) obtained after administration of intravenous iodinated contrast in soft tissue window centered at the level of the heart. There is aneurysmal dilatation of the right atrial appendage causing marked mass effect on the right ventricle, deforming its free wall (arrow). Note the 7.6 × 3 cm area of hypoenhancement/filling defect (asterisk) at the tip of the right atrial appendage, which could be the result of either slow flow with poor mixing of contrast or thrombus formation. Notice the layering of the iodinated contrast in the dependant portion of the right atrial appendage, more consistent with slow flow and poor mixing of contrast (arrowhead). The right coronary artery and acute marginal branch with surrounding epicardial fat are seen in the atrioventricular groove. A, RAA aneurysm; O, ostium of RAA; LV, left ventricle; RV, right ventricle; RCA, right coronary artery; AMB, acute marginal branch

Contrast-enhanced abdominal CT showed active sigmoid diverticulitis. The patient was put on bowel rest and was treated with intravenous hydration and antibiotics. Incidentally, on the superior aspect of the scan field, a massively enlarged RAA (10.0 × 6.0 cm) was noted causing marked mass effect on the right ventricle (RV) (Fig. 1). A large region of hypoenhancement (7.6 × 3.0 cm) was also identified at the tip of the RAA. Given the patient’s history of arrhythmia, the treating team was concerned about the presence of an intraluminal thrombus.

A cardiology consult was obtained and the patient was sent for echocardiography. Echocardiography similarly revealed an enlarged RAA that compressed the RV and contained an echogenic density at its tip. Right ventricular systolic pressure was 24 mmHg and the right ventricular inner diameter was mildly elevated at 3.6 cm. Doppler showed mild tricuspid and pulmonary valve insufficiency with normal flow rates. A normal left ventricular ejection fraction (55%) with concentric left ventricular hypertrophy was observed.

**Fig. 2:**
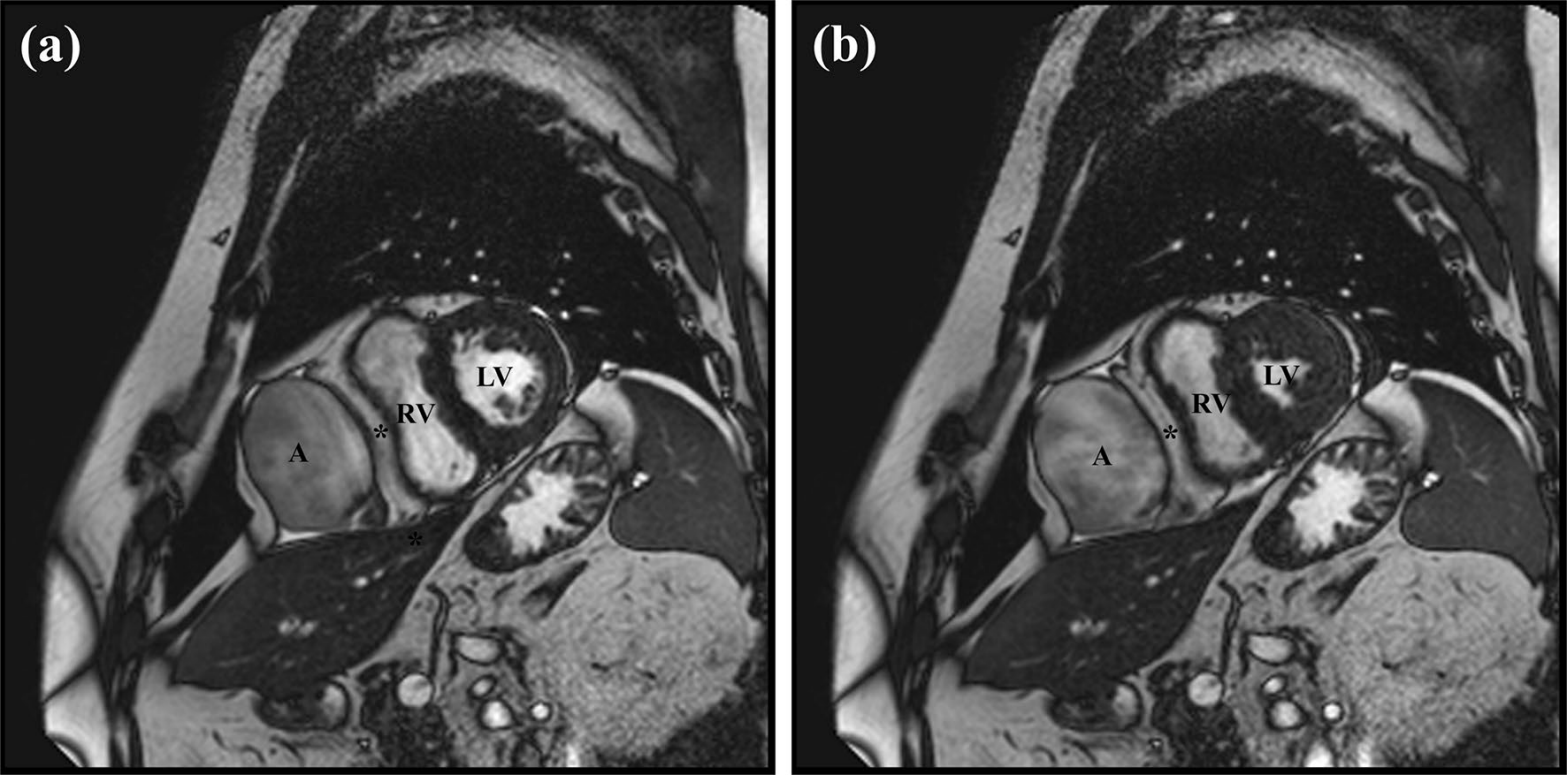
Balanced turbo field-echo short axial view of the right atrial appendage aneurysm. The well-defined, thin-walled right atrial appendage aneurysm can be seen compressing and deforming the right ventricle. Epicardial fat is seen between the right atrial appendage and the right ventricle (asterisk). The right atrial appendage aneurysm remains unchanged in size during **a** diastole and **b** systole, indicating an absence of contraction. The signal intensity in the aneurysm is lower than that of the blood pool in the right and left ventricles due to slow flow. A, RAA aneurysm; RV, right ventricle; LV, left ventricle

Cardiovascular magnetic resonance (CMR) (1.5 T, Philips Ingenia Imaging System^®^, Philips Healthcare^®^) imaging was subsequently performed. Balanced turbo field-echo (BTFE) short axis (sax) showed aneurysmal dilation of the RAA measuring 10.5 × 7.5 × 5.5 cm with a wide ostium of 3.6 × 3.6 cm. There was a lack of contractility of the aneurysm noted and evidence of significant compression of the right ventricular free wall during the cardiac cycle (see Fig. 2 or Additional file 1). Assessment of left and right global systolic functions using Simpson’s method in the short axis orientation revealed a normal left ventricular ejection fraction of 62% and a normal right ventricular ejection fraction of 57%.

**Fig. 3:**
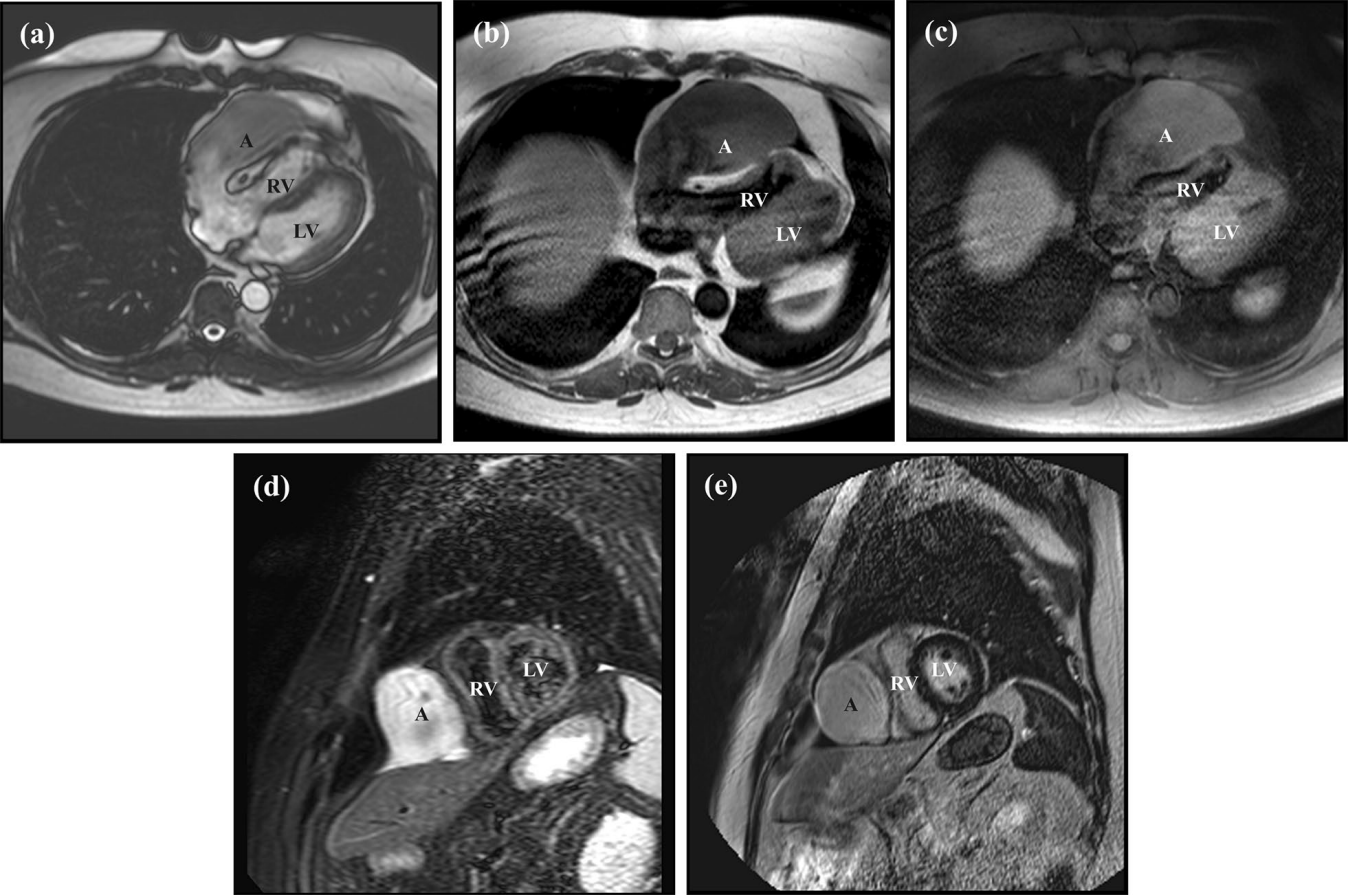
Additional cardiovascular magnetic resonance images centered at the right atrial appendage aneurysm. On balanced fast field echo using SENSE technique in axial orientation (**a**), the intraluminal signal of the RAA aneurysm is lower than that of the bright blood pool in the right and left ventricles, except for the area of high signal intensity at the right atrial appendage tip. On T1 spin echo in axial orientation without fat saturation (**b**) and with fat saturation (**c**) the blood in the right atrial appendage has intermediate signal intensity and does not follow the dark blood signal in the right ventricle. On the T2 short-tau inversion relaxation sax image (**d**), there is high signal intensity in the right atrial appendage in contrast to the blood pool in the ventricles on the post-contrast sax image (**e**), the signal in the RAA is similar to the blood pool in the right ventricle and left ventricle. There is no wall or intraluminal enhancing component. A, RAA aneurysm; RV, right ventricle; LV, left ventricle

Multiple CMR sequences showed a difference in signal between the blood pool contained within the RAA and the rest of the cardiac chambers (Fig. 3). Balanced fast field echo using SENSE technique (sB-FFE) showed that the contents of the RAA aneurysm was of lower signal intensity than the bright blood signal in the ventricles. T1-spin echo (T1-SE) images obtained without fat saturation demonstrated that the contents of the RAA aneurysm were of intermediate signal intensity as opposed to the dark blood signal in the RV. The signal intensity did not change with fat saturation. T2-weighted short-tau inversion relaxation (STIR) showed high signal intensity in the RAA in contrast to the dark blood pool in the ventricles. Finally, post-contrast sax images showed that the signal in RAA aneurysm was similar to that of the ventricular blood pools.

**Fig. 4:**
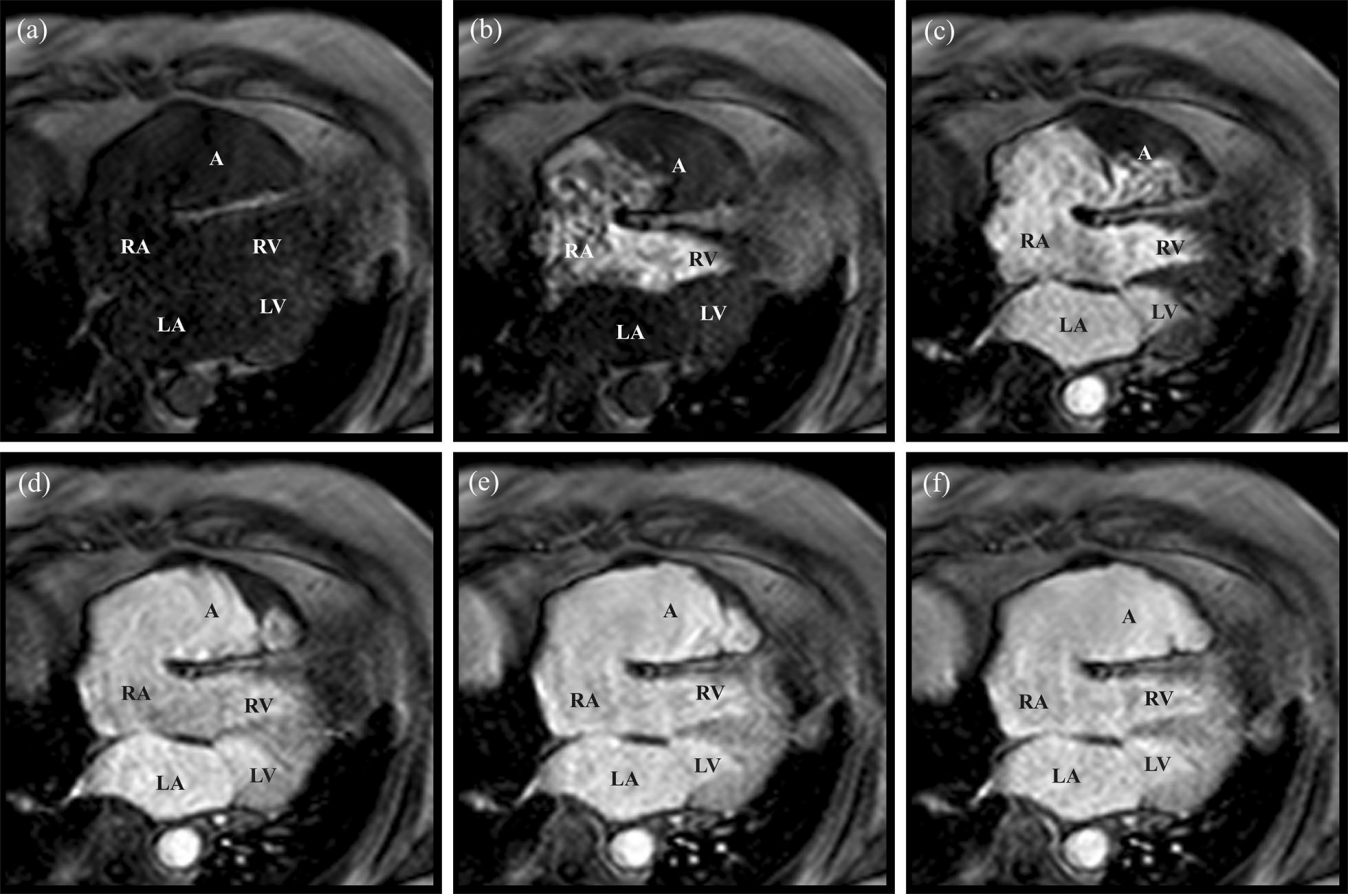
Axial static images of the first pass perfusion cine taken with administration intravenous gadolinium. Prior to injection of contrast material, **a** the right atrial appendage aneurysm can be visualized compressing the right ventricle. After administration of 25 mL gadolinium IV contrast agent (Multihance^®^), there is sequential opacification of the right (**b**) and the left (**c**) cardiac chambers. Afterwards, there is slow, progressive contrast opacification of the stagnant blood pool in the right atrial appendage aneurysm until complete contrast opacification of the aneurysm (**d**–**f**). This indicates that the original area of hypoenhancement found on computed tomography was in fact due to slow flow, not a thrombus. A, RAA aneurysm; LA, left atrium; LV, left ventricle; RA, right atrium; RV, right ventricle

First-pass perfusion axial images were obtained following the administration of 25 mL of IV gadolinium contrast agent (Multihance^**®**^) (Additional file 2 and Fig. 4). The cine showed progressive mixing of the RAA aneurysm contents with contrast material until complete opacification. The signal abnormality on CT, echocardiography, and CMR was therefore concluded to have been caused by slow-mixing blood. Given the patient’s advanced age and the lack thrombotic disease, the patient was managed medically with anticoagulation.

## Discussions

RAA aneurysms are rare clinical entities. In the present case, gadolinium-enhanced CMR was used to exclude an aneurysmal thrombosis and orient the treating team towards medical management. To the best of the authors’ knowledge, this is first published case where CMR was used to direct therapy in such a context.

Echocardiography is the initial investigation of choice in the workup of cardiac abnormalities. It is inexpensive, widely available and minimally invasive. Echocardiography plays a particularly important role in the pediatric population, where the radiation exposure of CT scans and the long scan times of CMR are of particular concern [9]. Indeed, agitated saline echocardiography has been used to differentiate RAA aneurysms from pericardial effusions in the neonate [7] and fetal ultrasound has been used to detect RAA aneurysms in the prenatal period [2, 9, 16]. Compared to CT and CMR, however, echocardiography has a poorer ability to differentiate RAA aneurysms from similarly-appearing anomalies [4] such as pericardial cysts [6], Ebstein’s anomaly [9], cor triatriatum [8], epicardial and pericardial fat lesions, and coronary aneurysms.

CT and CMR are both highly specific for the detection of RAA aneurysms [4]. CT offers the benefit of a faster capture time, but it exposes patients to ionizing radiation and nephrotoxic contrast agents. Filling defects can be assessed on CT with delayed phase scanning 1–2 min after contrast administration, but this study is difficult to protocol in advance unless the operator is already aware of the defect.

CMR offers superior anatomical resolution and the ability to observe delayed blood mixing over time with cine imaging. CMR also has the ability to evaluate the function of the RV and to observe any mass effect exerted on the heart during the cardiac cycle. Presently, the use of CMR to assess RAA aneurysms in the literature has been limited; CMR was used in only 8 of 22 published English-language case reports (see Table 2 for a summary of published findings). This is likely due to the relatively recent emergence of the technology and its limited availability. Regardless, CMR can be invaluable in characterizing such cardiac abnormalities.

**Table 2:** Literature review of cardiovascular magnetic resonance used in the assessment of right atrial appendage aneurysms

Paper	CMR parameters^a^	Description of aneurysm
Contreras et al. [18]	Steady-state free precession	None provided
Corti et al. [12]	Turbo field-echo movie reconstruction	Dimensions: 7 × 5 × 3.5 cm 2.8 cm ostium Right ventricular compression Systo-diastolic filling of the mass consistent with right atrial filling
Duran et al. [21]	Not specified	Dimensions: 2.5 × 1.5 cm over the tricuspid valve Neck: 11 mm No thrombus
Guaricci et al. [10]	Not specified	Above tricuspid valve, overlapping with the RV and outflow tract with pulmonary artery
Gulati et al. [15]	Cine imaging using balanced steady-state free precession sequence	Mild tricuspid regurgitation Noncontractile, smooth-walled, pyramidal RAA extending anterior to the body of the right atrium Partially separated from the main atrial chamber by a membrane Right atrial volume 118 mL/m^2^ Mild distortion of the RV but no compression
Le Ven et al. [22]	Not specified	None provided
Sondhi et al. [17]	Not specified	Magnetic resonance imaging of the heart and vessels confirmed the connection of the aneurysm to the RAA and also showed slight compression of the descending thoracic aorta by aneurysm
Xu et al. [8]	None described	None provided

^a^All images contrast-enhanced

In present case, the treating team opted to pursue medical management once the area of hypoenhancement within the RAA aneurysm was characterized as slow flow by CMR. This conservative approach has been supported in the literature for patients who are asymptomatic and elderly [1, 6, 8]. Conversely, in patients who are younger, symptomatic, or who have evidence of thrombosis, surgical excision appears to be the preferred therapy [2, 5, 12–15]; a Cox-maze procedure may also be performed to treat concomitant arrhythmias [10, 14]. The rarity of RAA aneurysms makes it difficult to determine whether such an intervention confers a survival benefit, however [20].

The interaction of RAA aneurysms with other comorbidities merits mention. The patient in the present case also suffered from diverticulitis, a condition which may be treated medically or surgically. Such a scenario calls into question how other diseases should be managed in the context of RAA aneurysms, especially those that require surgery. Unfortunately, there is a paucity of data available in the literature to guide clinicians. The presence of a RAA aneurysm in and of itself does not appear to be a contraindication to surgery. However, if the aneurysm is large enough to affect cardiac output, contribute to outflow tract obstruction, or cause valvular regurgitation [9], then these comorbidities must be taken into consideration. For example, if the patient is preload dependent due to the impairment of ventricular function, this should be considered in the patient’s perioperative fluid management. If the patient presents in heart failure, inotropes and a monitored setting should be considered to maintain adequate post-operative tissue perfusion. In the present case, given the patient’s preserved biventricular function and minimal regurgitation, few peri-operative modifications would have been necessary. A pre-operative medical consultation would nonetheless have been recommended.

## Conclusions

In conclusion, we present the use of CMR to characterize a giant RAA aneurysm containing an area of hypoenhancement on CT that was concerning for a massive thrombus. Multiplanar, multisequential gadolinium-enhanced CMR—including first-pass perfusion cine imaging—was used to characterize this area as slow flow and the patient was managed medically. Despite the rarity of this condition, future studies should be directed towards determining whether surgical management is of benefit compared to medical management.

## Additional files

**Additional file 1.** Balanced turbo field-echo cine in short axis view. The size and position of the right atrial appendage aneurysm is shown in relation to the right ventricle. Note how the right atrial appendage aneurysm compresses and deforms the right ventricle throughout the cardiac cycle.

**Additional file 2.** First pass perfusion cine of the right atrial appendage aneurysm in axial plane following the administration of IV gadolinium. Following the administration of 25 mL of IV gadolinium contrast agent (Multihance^®^), there is progressive and complete enhancement of the RAA aneurysm. This indicates that the area of hypoenhancement found on contrast-enhanced computed tomography was slow-flowing blood, rather than a thrombus.

## Abbreviations

AMB: acute marginal branch
BTFE: balanced turbo field-echo
CMR: cardiac magnetic resonance
CT: computed tomography
LA: left atrium
LV: left ventricle
LVEF: left ventricular ejection fraction
MRI: magnetic resonance imaging
O: ostium
RA: right atrium
RAA: right atrial appendage
RCA: right coronary artery
RV: right ventricle
sax: short axis
SB-FFE: balanced fast field echo using SENSE technique
STIR: short-tau inversion recovery
T1-SE: T1 spin echo
